# Biomarkers of subclinical cholestasis predict cancer immunotherapy outcomes

**DOI:** 10.1080/2162402X.2026.2748140

**Published:** 2026-10-15

**Authors:** Anne-Laure Mallard de La Varende, Marine Fidelle, Guido Kroemer, Laurence Zitvogel

**Affiliations:** a Gustave Roussy (GRCC), ClinicObiome, Inserm UMR1367, Microbiota and Mucosal Immunity for Cancer Immunotherapy, Université Paris-Saclay, Villejuif, France; b Centre de Recherche des Cordeliers, INSERM U1138, Équipe Labellisée – Ligue Nationale Contre le Cancer, Université Paris Cité, Sorbonne Université, Paris, France; c INSERM US23, Analyse moléculaire, modélisation et imagerie de la maladie Cancéreuse, Plateformes de Métabolomique et de Criblage Cellulaire Haut Débit, Université Paris-Saclay, Villejuif, France; d Institut Du Cancer Paris CARPEM, Department of Biology, Hôpital Européen Georges Pompidou, AP-HP, Paris, France

**Keywords:** Cancer, PD-1, immune checkpoint blockade, bile acids, MAdCAM-1, antibiotics, gut microbiota, microbiome, cholestasis

## Abstract

In a recent paper published in Cancer Cell, we identified subclinical cholestasis as an accessible imprint of gut dysbiosis predicting resistance to immune checkpoint blockade. Our work connects *γ*-glutamyl transferase, bile-acid remodeling, and MAdCAM-1 loss to compromised antitumor immunity, opening avenues for novel biomarker-guided microbiota interventions.

Immune checkpoint blockade (ICB) can induce durable tumor control, yet most patients either fail to respond or ultimately relapse. Tumor genomics, PD-L1 expression, and immune infiltration explain only part of this heterogeneity. The gut microbiota adds another layer: antibiotics and dysbiotic microbial configurations repeatedly associated with inferior ICB efficacy, but stool metagenomics remains difficult to standardize, geographically sensitive, and remote from routine oncology.^1^ ^,^ ^2^ The crucial challenge is therefore not merely to catalog microorganisms, but also to identify host-readable traces of dysbiosis that are mechanistically informative, analytically robust, and clinically deployable. A blood-based signature would solve a problem: it could be measured repeatedly, compared across centers, and incorporated into pretreatment workflows without requiring the complex logistics of fecal sampling, microbial DNA sequencing, and lengthy bioinformatic data deconvolution.

In a recent paper, we have connected this problem to an unexpected biochemical phenotype: subclinical cholestasis.^3^ In antibiotic-treated mice bearing extraintestinal tumors, dysbiosis depleted secondary bile acids, including deoxycholic acid (DCA), while favoring a systemic surge in tauro-conjugated primary bile acids, including tauro-muricholic acid (Tα, *β*, *ω* MCA) and tauro-chenodeoxycholic acid (TCDCA). Gavage with *Enterocloster clostridioformis* partly phenocopied this metabolic shift. Conversely, fecal microbiota transplantation (FMT), the ileal farnesoid X receptor (FXR) agonist fexaramine, or glycodeoxycholic acid (GDCA) restored a more favorable bile-acid configuration and responsiveness to PD-1 blockade. These interventions convert association into a coherent causal chain: (i) dysbiosis perturbs enterohepatic metabolism; (ii) altered bile acids reprogram immunity; and (iii) immune dysfunction compromises tumor control. The concordance between antibiotic exposure, a defined bacterial genus, experimental cholestasis and three corrective interventions provides triangulation across microbial, metabolic and immune compartments.^3^

The immunological hinge is mucosal addressin cell adhesion molecule-1 (MAdCAM-1), an ileal vascular checkpoint retaining α4β7-positive enterotropic lymphocytes. Previous work showed that *Enterocloster*-driven MAdCAM-1 downregulation releases immunosuppressive T helper 17-like regulatory cells toward tumors.^4^ Our new study places bile acids upstream of this trafficking defect. Oral administration of GDCA increased ileal MAdCAM-1 expression, restrained dissemination of enterotropic regulatory T cells, and curtailed exhaustion of tumor-infiltrating lymphocytes. Its activity disappeared in *Madcam1*-deficient mice.^3^ Thus, the gut‒liver‒tumor axis is not a merely metaphorical connection but constitutes an experimentally interruptible circuit, consistent with prior evidence that bile-acid synthesis can directly inhibit tumor-specific T lymphocytes in liver cancer.^5^ Importantly, direct induction of cholestasis by bile duct ligation, which elevates circulating liver enzymes and alters systemic bile acid profiles by disrupting the enterohepatic cycle, was sufficient to abrogate the therapeutic efficacy of immunotherapy.^3^

The biomarker implications are substantial (Figure 1). First, *γ*-glutamyl transferase (γGT), an inexpensive and universally available laboratory measurement, transforms a complex ecological disturbance into a potentially actionable clinical flag. Baseline γGT at least twice the sex-specific upper limit of normal identified approximately one-quarter of patients and was associated with inferior progression-free and/or overall survival across six cohorts of 600 cancer patients encompassing several tumor types and therapeutic regimens.^1^ Earlier observations in ICB-treated melanoma and renal-cell carcinoma now acquire a mechanistic framework rather than remaining isolated clinical correlations.^6^ Importantly, γGT correlated positively with tauro-conjugated bile acids and negatively with soluble MAdCAM-1 (sMAdCAM-1), linking routine biochemistry to microbial metabolism and intestinal immune trafficking.^3^

**Figure 1.**
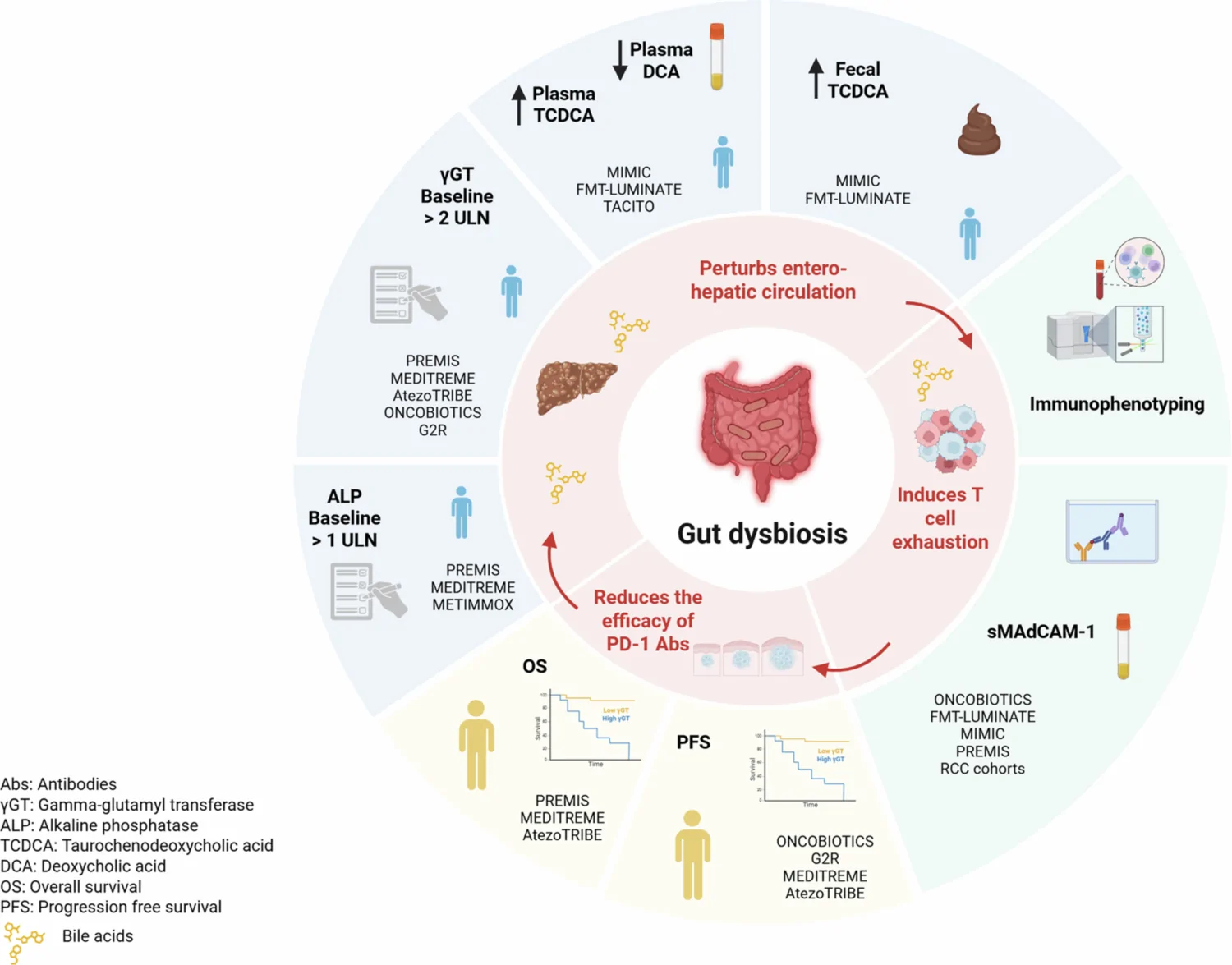
Cholestasis-associated circulating biomarkers linked to cancer immunotherapy outcome.

Second, our study suggests a tiered biomarker architecture rather than a single privileged analyte.^3^ γGT and alkaline phosphatase constitute a scalable first layer; mass-spectrometric quantification of TCDCA, TCA, DCA, GDCA, and the glyco-to-tauro-conjugated bile-acid ratio provides metabolic resolution; sMAdCAM-1 adds mechanistic proximity to the intestinal vascular checkpoint. Low circulating sMAdCAM-1 has already been associated with adverse outcomes across patients with lung, bladder, kidney, and colorectal cancers,^4^ ^,^ ^7^ and has subsequently shown independent prognostic value in patients with metastatic renal-cell carcinoma.^8^ Longitudinal measurements may be particularly revealing: TCDCA tends to rise in nonresponders, whereas FMT-containing regimens promote descending TCDCA trajectories.^3^ ^,^ ^9^ ^,^ ^10^ Together, these markers could identify dysbiosis without demanding immediate stool sequencing while reserving metagenomics for biological refinement and intervention selection.

This promise nevertheless requires terminological and statistical discipline. An isolated γGT elevation is a surrogate of cholestasis, not its diagnosis, and can reflect liver metastases, alcohol consumption, metabolic liver disease, biliary obstruction, medications, or immune-related hepatotoxicity. The multivariable analyses addressed several major prognostic factors but could not exhaust these alternatives. Moreover, a biomarker associated with outcome under ICB is not automatically predictive of ICB benefit. The absence of a clear γGT effect in the chemotherapy-only AtezoTRIBE arm is provocative, but a treatment-by-biomarker interaction, prospectively specified and adequately powered, is needed to distinguish the prediction from general prognosis. Threshold transportability across laboratories and populations also remains to be established.

Prospective validation should therefore compare the cholestatic signature with established variables, including performance status, tumor burden, liver metastases, PD-L1, neutrophil-to-lymphocyte ratio, antibiotics, and concomitant medication. Prespecified cutoffs, blinded assays, calibration analysis, decision curves, and serial sampling are indispensable. Equally important will be testing whether a composite score materially outperforms γGT alone. Biomarker discovery becomes clinically meaningful only when it changes classification, treatment, or monitoring – not when it merely adds another statistically significant correlate.

The most optimistic interpretation is also the most testable. Subclinical cholestasis may identify an immunoresistant state that is reversible rather than irrevocably tumor intrinsic. FMT has already shown feasibility and activity alongside immunotherapy,^9^ ^,^ ^10^ while GDCA and intestinal FXR manipulation offer metabolically defined alternatives. Yet the human data do not currently justify treating an elevated γGT value with GDCA or FMT. Biomarker-enriched trials must demonstrate that correcting the bile-acid–MAdCAM-1 axis improves ICB outcome. If successful, routine liver biochemistry will have acquired an unexpected function: revealing a distant intestinal defect and guiding its repair. This sequence is exemplary: start with microbial ecology, identify a circulating metabolic disturbance, connect it to a tissue checkpoint, reduce the signal to routine analytes, and finally nominate corrective interventions. Few microbiome studies traverse the distance from taxonomic association to a testable clinical strategy.

## Data Availability

This paper does not contain any original data.
